# The Substitution Evil

**Published:** 1901-01

**Authors:** 


THE SUBSTITUTION EVIL.—No greater wrong is perpetrated upon the physician than to have his dispensing druggist, in whom, perhaps, he has every confidence, in putting up a prescription, substitute something of his own compounding for some standard, well known, well tested and approved pharmaceutical preparation. The medical press should with one voice protest against the practice, and urge their readers, the rank and file of the profession—the working doctors (and, as Dr. Bacon Saunders said of them, "they are the salt of the earth"), to suspect all druggists, where at any time expected results from some preparation have not been observed; and to carefully investigate and see if they are being imposed upon by the substitution of something "just as good." If any dispenser is caught in the nefarious practice, all the brethren should be warned against him, and their patronage should be transferred to an honest druggist. That is about the only way the practice can be broken up.

Not only is it a wrong inflicted upon the doctor—he is deceived and cheated by having useless tools put in his hands, when he has expected those upon which he knows he can rely; but it is an outrage upon common decency and honesty; it is a cruel wrong to the patient and in a crisis may be the deciding thing in his fate; it may mean death, where if the doctor's orders are honestly filled, the patient would—might, at least—recover. It is robbery, too, of the honest manufacturers, whose goods have stood the critical tests of experience in the hands of capable clinicians, and are stamped with the approval of the medical men. It is unfortunate that this practice is not made a felony, it deserves to be.

Of course, the JOURNAL cannot specify any of the many valuable pharmaceuticals that are being counterfeited, or for which worthless stuff is being substituted in the doctor's prescription; it would seem invidious, it would be unjust to other just as deserving and valuable products. Our readers all know them. When a doctor prescribes a tried and approved preparation, he should make it his business to *see that the patient gets it.*

Of course nothing in this can touch the honest and conscientious dispensing druggist.

